# The Effects of White Wine and Ethanol Consumption on the Proliferative Phase of Repair After a Surgically Induced Myocardial Infarction in Rats

**DOI:** 10.3390/nu17040699

**Published:** 2025-02-15

**Authors:** Danica Boban, Ivica Grković, Ana Marija Dželalija, Diana Gujinović, Ivana Mudnić, Mladen Boban

**Affiliations:** 1 Department of Anatomy, Histology and Embryology, University of Split School of Medicine, Šoltanska 2A, 21000 Split, Croatia; danica.boban@mefst.hr (D.B.); ivica.grkovic@mefst.hr (I.G.); 2 Department of Basic and Clinical Pharmacology, University of Split School of Medicine, Šoltanska 2A, 21000 Split, Croatia; ana.marija.milat@mefst.hr (A.M.D.); diana.juric@mefst.hr (D.G.); ivana.mudnic@mefst.hr (I.M.)

**Keywords:** CD163, CD68, macrophages, white wine, myocardial infarction, rat

## Abstract

**Background:** Our recent findings, of the highest survival rate of animals that consumed moderate amounts of white wine for four weeks prior to surgically induced myocardial infarction by the ligation of the left anterior descending artery, prompted us to investigate the inflammatory aspects of the post-infarction healing process. In order to examine whether the effects of wine consumption differ from that of ethanol, experimental animals were randomized into three groups: white wine, 13% *v*/*v* ethanol/water or water-only controls. **Methods:** Hearts for immunohistochemical analysis were collected from animals that survived 96 h after infarction, consumed no less than 8 mL of white wine or ethanol/water solution per day and had transmural infarcts of comparable sizes. After accounting for all of the above criteria, the final number of animals was seven per group. Tissue slices were stained with a pan-macrophage marker CD68 and an anti-inflammatory macrophage marker CD163 to investigate macrophage polarization that is crucial for the inflammatory aspects of post-infarction healing. Immunofluorescent imaging was performed on four zones surrounding the infarcted area with detritus: subepicardial, subendocardial and two peri-infarct zones. **Results:** The largest CD163/CD68 ratios for comparable volumes of alcohol consumption were observed in the wine group in all zones. CD163/CD68 ratios decreased in both the ethanol and wine group as the average amount of alcoholic beverage consumed by the animals increased. **Conclusions:** Our results indicate that non-alcoholic constituents of white wine contribute to its superior effects in the favorable modulation of post-infarction inflammation and healing processes relative to that of ethanol alone.

## 1. Introduction

Cardiovascular diseases (CVDs) are the leading cause of death globally, and acute myocardial infarction (MI) is the main contributor to the total death toll [1]. The moderate consumption of alcohol, particularly in the form of red wine and in the context of healthy diet and lifestyle, has been associated with a reduced CVD morbidity and mortality risk [2,3,4,5,6,7,8,9,10]. Indeed, moderate wine consumption is a typical and important component of the Mediterranean diet [11,12], which is considered to be one of the healthiest diets in the world [13]. The WHO has identified the Mediterranean-like dietary patterns as beneficial in preventing and controlling diet-related non-communicable diseases, which are the leading cause of premature death [14]. Moreover, the regular moderate consumption of alcohol, particularly red wine, has been identified as one of nine lifestyle elements that are common denominators of the longest living people from different geographic areas around the world [15]. Epidemiological and clinical findings of an inverse association between regular and moderate wine consumption and cardiovascular risk have provoked numerous studies on the mechanisms of the beneficial biological effects of wine and its constituents. Despite the increasing production and popularity of white wines among consumers [16], most studies have focused on the cardioprotective effects of red wines. This is mainly due to their much higher content of non-alcoholic bioactive compounds, primarily polyphenols, relative to white wines [17]. In our recent study, we showed the highest survival rate of animals consuming moderate amounts of white wine for four weeks prior to a surgically induced MI relative to ethanol and water drinking rats [18]. The quality and outcome of post-MI healing and scar tissue formation is critically dependent on a balanced inflammatory response [19,20]. Macrophages are significant regulators and participants in that process [19]. The polarization of macrophages towards a reparative M2 phenotype over the pro-inflammatory M1 phenotype is associated with the improved healing of infarcted myocardium [19,20,21,22,23]. In order to investigate the nature of interplaying roles of ethanol and wine phenolics on modulation of inflammation and macrophage polarization [24,25,26], we examined and compared the effects of white wine and ethanol/water solution on the inflammatory aspects of the post-MI healing process. One of the most robust tools for detecting the immunophenotype switch in macrophage population is the immunohistochemical detection of the cluster of differentiation (CD) antigens. Using double immunohistochemistry for CD68, as a ‘pan-macrophage’ marker, and CD163 as a M2 marker, we were able to perform the differentiation of two subpopulations of macrophages [19]. The information obtained using those two markers was combined into a CD163/CD68 ratio, providing insight into the fraction of M2 macrophages within the total macrophage population. Furthermore, we examined how the volumes of consumption of the tested alcoholic beverages for four weeks prior to the induction of MI correlated with the kinetics of these parameters in the post-infarction myocardial tissues.

## 2. Materials and Methods

### 2.1. Ethical Considerations

All procedures and experimental protocols were in accordance with the European Convention on Animal Protection and Guidelines on Research Animal Use and were approved by the Ethics Committee of the University of Split School of Medicine and by the Ethics Committee of the Ministry of Agriculture of the Republic of Croatia (No. 525-10/0255-16-7).

### 2.2. Animal Model and Experimental Design

**Figure 1:**
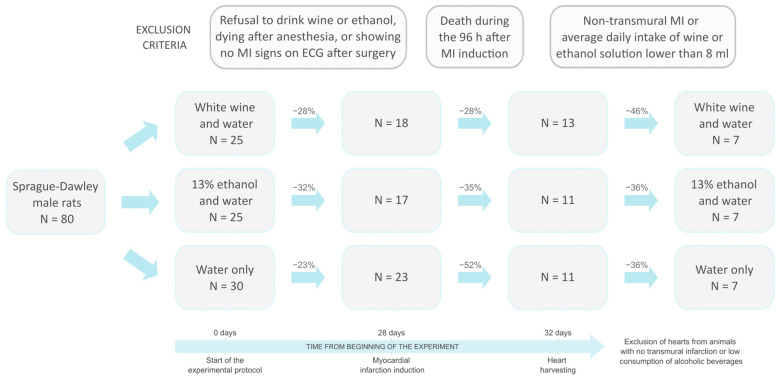
Flow diagram showing exclusion criteria and the number of animals remained in each step of the study protocol. The numbers above the blue arrows denote the dropout percentages after applying corresponding exclusion criteria.

Initially, 80 male Sprague Dawley (SD) rats, aged 6 weeks, weighing 145–150 g, were used in this study. The exact numbers of animals that fulfilled the inclusion/exclusion criteria through the various steps of this experiment can be seen in the flow diagram (Figure 1).

The rats were raised in the Experimental Animal Facility of the University of Split in a temperature-controlled environment (23 ± 2 °C), adhering to a 12 h light/dark cycle. Animals were housed individually in solid-bottom plastic cages with sawdust bedding and fed a standard diet consisting of rat chow (4RF21 GLP, Mucedola srl, Milano, Italy) and were weighed weekly to monitor growth and health status throughout the 4-week study period. Experimental animals were randomly assigned to one of three groups: water only, white wine or 13% *v*/*v* ethanol/water solution. The white wine group drank Graševina, from the Krauthaker winery in Croatia, containing 13% alcohol. The detailed analyses of biochemical properties and the phenolic constituents of this wine were reported in our previous study [27]. Animals in the water-only group had ad libitum (available 24 h a day) access to fresh tap water throughout the day. The white wine and ethanol solution groups were provided with their respective beverages ad libitum while also having access to tap water for 6 h daily. All beverages were supplied in small pet containers with a leak-proof nozzle (Ferplast SmallPet Sippy Water Bottle, Vicenza, Italy). This protocol ensured that all animals received fresh beverages consistently, allowing for the accurate daily measurement of fluid intake over the 4-week period prior to the induction of the MI. Animals that refused to consume the white wine or the ethanol/water solution were excluded from the study. 

### 2.3. Surgical Procedure and ECG Recording

**Figure 2:**
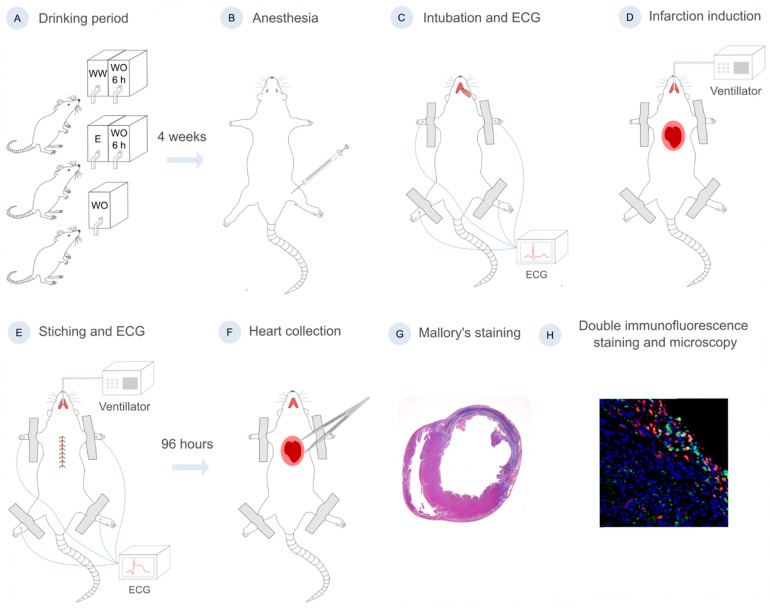
A flow chart of the study showing: (**A**) The distribution of animals into 3 experimental groups (WW—white wine group; E—13% ethanol/water solution drinking group; WO—water-only drinking group); (**B**) Intramuscular anesthetic application using right hamstring muscle; (**C**,**E**) pre- and postoperative ECG recording; (**D**) MI induction by ligation of LAD; (**F**) harvesting following 96 h survival; (**G**,**H**) histological and double immunohistochemical staining.

All animals were anesthetized and then the MI was surgically induced through the ligation of the left anterior descending (LAD) coronary artery. The methodology for the “transdiaphragmatic approach” performed in the surgical procedure has been detailed in a previous publication by our group [28]. To confirm the successful induction of the MI, electrocardiogram (ECG) recordings were used. The rat, placed in a supine position on a heated surgical table, was connected to four needle electrodes, one for each limb. ECG recordings (Cardioline Delta 1, Trento, Italy) were performed before the initial abdominal incision, at the beginning of surgery, and following the final suturing that completed the surgical procedure. The broadening of the QS complex, the elevation of the ST segment, and T-wave inversion were regarded as signs of the successful induction of myocardial ischemia. Animals that did not show clear signs of ischemia on ECG recordings and those that did not survive 96 h post-MI were excluded from the study. Those animals that did survive were euthanized, and their hearts were extracted and immersion-fixed. A scheme of the study with each experimental step is shown in Figure 2.

### 2.4. Tissue Processing

After fixation in Zamboni’s fixative (4% paraformaldehyde and 0.2% picric acid in 0.1 M PBS at pH 7.4) for 3 days, the ventricles were transversely sectioned into three segments. The initial cut was made 4 mm below the left auricle margin, followed by a second cut 4 mm beneath the first. The tissue samples were then embedded in paraffin wax, serially sectioned, and mounted on glass slides.

**Figure 3:**
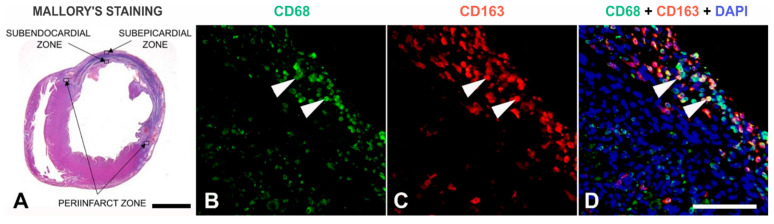
A cross-section of the heart (Mallory’s staining) showing a large infarcted area of the anterior wall of the left ventricle with four zones that have been analyzed (**A**). CD68-immunoreactivity (green) (**B**) and CD163-immunoreactivity (red) (**C**) of the subepicardial zone showing labeled macrophages. When individual images obtained using different filters were merged together and overlayed on top of the DAPI staining signal, the colocalization could be clearly detected (white arrowheads) (**D**). Scale bars: (**A**) = 2.5 mm; (**B**–**D**) = 100 μm.

Histological examination using Mallory’s trichrome staining was used to confirm the transmural infarction. The significant necrosis of cardiac tissue, the loss of cardiomyocytes, an influx of inflammatory cells (such as neutrophils and macrophages), the thinning of the muscular wall of the left ventricle and the beginnings of granulation tissue formation were observed (Figure 2G and Figure 3A). After Mallory’s trichrome staining, 4 zones of interest for analysis were determined (Figure 3A).

### 2.5. Immunofluorescence Staining

Prior to staining, sections underwent deparaffinization, followed by heat-induced epitope retrieval using a steamer with preheated sodium citrate buffer (pH = 6.0) for 30 min. To minimize non-specific binding, a Protein Block solution (ab64226, Abcam, Cambridge, UK) was applied for 30 min. The sections were then incubated overnight in a humified chamber with primary antibodies: recombinant monoclonal rabbit Anti-CD163 antibody (ab182422, Abcam, Cambridge, UK, diluted at 1:250) and monoclonal mouse Anti-CD68 antibody (ab31630, Abcam, Cambridge, UK, diluted at 1:250). After washing with phosphate-buffered saline (PBS), the sections were incubated for 1.5 h with a combination of species-specific secondary antibodies, namely Alexa Fluor 594 goat anti-rabbit (ab150084) and Alexa Fluor 488 goat anti-mouse (ab150117), both manufactured by Abcam (Cambridge, UK) and diluted at 1:300. Following another wash in PBS, the sections were stained with 4′6-diamidino-2-phenylindole (DAPI) to visualize nuclei. The slides were air-dried and mounted in mounting media (Immuno-mount, Shandon, Pittsburgh, PA, USA). Finally, the sections stained using Mallory’s staining were photographed using a Canon PowerShot A480 (Canon, Tokyo, Japan) photographic camera, set to super-macro settings. Sections stained with antibodies were visualized and photographed using a Nikon DS-Ri2 camera (Nikon Corporation, Tokyo, Japan) with NIS-Elements F software (version 5. 22. 00) and a light microscope Olympus BX51 (Olympus, Tokyo, Japan) equipped with an Olympus DP71 camera.

### 2.6. Quantification

Four representative assessment zones were established in relation to the infarcted area: two peri-infarct zones on each end of the infarcted area, the area between the infarct and the endocardial surface (subendocardial zone), and the area between the infarct and the epicardial surface of the left ventricle (subepicardial zone). A cross-section of the heart showing the abovementioned zones is presented in Figure 3A.

In each of the listed zones, 4 non-overlapping neighboring fields were captured for the analysis, at 40× magnification. For every field, four photographs were taken through three different filters, namely green (Alexa Fluor 488)—Figure 3B; red (Alexa Fluor 594)—Figure 3C; and blue (DAPI)—visible in Figure 3D, which shows all filters merged. This enabled us to co-localize CD163 and CD68 with DAPI on every field that was analyzed. Microphotographs were then analyzed using ImageJ v. 1.54f computer program (NIH, Bethesda, MD, USA). The quantification of the total number of positive cells for macrophage markers per field was performed using the “Multipoint tool”, where positive cells were counted as those showing overlap between DAPI and the signal of the specific primary antibody. The cell density for each marker was calculated based on the area of the field after excluding healthy tissue regions and artifacts from the image and then averaged for each zone.

### 2.7. Statistical/ Data Analysis

The R programming language for statistical computing version 4.4.2 was used for all statistical analysis and data visualization. The data were presented as a mean ± standard error. The normality of distributions was tested by visualizing the data, inspecting the Q-Q plots and using the Shapiro–Wilk test. The comparisons of means of three groups was performed using the one-way ANOVA test, and the comparisons of two groups used the *t*-test for independent samples. The estimated marginal means were derived from a linear model fitted to the data, accounting for the group and zone variables, as well as the consumed amount of alcoholic beverage. *p*-values lower than 0.05 were considered statistically significant. Abbreviations with the following meanings were used to declare the significance level in figures and tables: *** *p* < 0.001, ** *p* ≥ 0.001 and *p* < 0.01, * *p* ≥ 0.01 and *p* < 0.05, (not significant) n.s. *p* ≥ 0.05.

## 3. Results

The study involved three experimental groups: the white wine group, the ethanol/water solution group and the water-only drinking group. There was no statistically significant difference in the final body mass at surgery time between the three groups (*p* = 0.54, one-way ANOVA test). The average amount of daily alcoholic beverage intake also did not differ significantly between the groups (*p* = 0.11, Student’s two-sample *t*-test). The amount of water consumed was significantly higher in the water group, but no significant difference was observed when the ethanol and wine groups were compared (*p* = 0.21, Student’s two-sample *t*-test) (Table 1).

To determine whether there are differences between the effects of white wine, ethanol and water on the inflammatory aspects of the post-infarction healing process, three values were determined for each zone surrounding the infarcted heart tissue: the density of the CD163- and CD68-positive macrophages and the ratio of the number of CD163- and CD68-positive macrophages.

**Figure 4:**
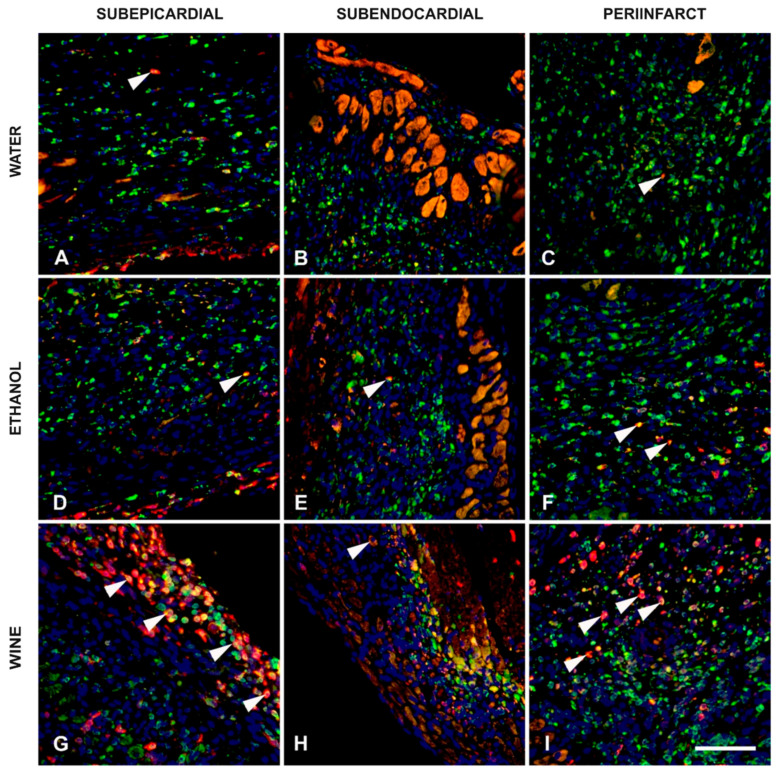
Display of macrophage densities across analyzed zones. CD68-immunoreactivity is shown in green, CD163 immunoreactivity in red and DAPI nuclei staining in blue. Images (**A**–**C**) show the merged immunofluorescence of the water-only experimental group for three zones (subepicardial in (**A**), subendocardial in (**B**) and peri-infarct zone in (**C**)). Similarly, three zones were displayed for the ethanol (images D–F) and for white wine groups (images G–I). Different densities of colocalization are noticeable (arrowheads). All images are of the same magnification, scale bar = 75 μm.

**Figure 5:**
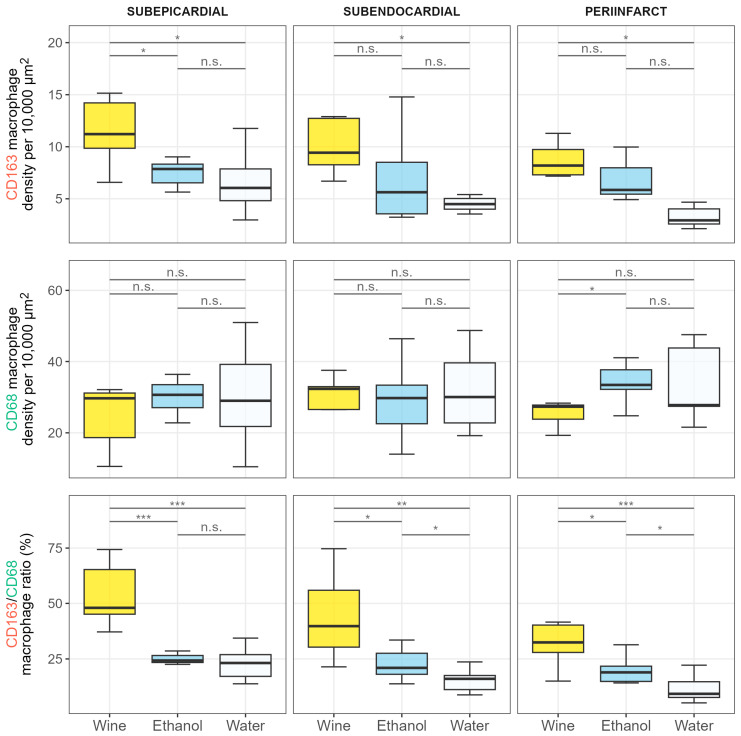
Comparison of macrophage densities and ratios between groups for different macrophage types across analyzed zones. The horizontal lines at the top of the plots and the text above them indicate the significance of Student’s two-sample *t*-tests. *** *p* < 0.001; ** *p* ≥ 0.001 and *p* < 0.01; * *p* ≥ 0.01 and *p* < 0.05; n.s. *p* ≥ 0.05.

For CD163 macrophages, there was a significant difference between the densities in the wine and water drinking groups within all zones, while the difference was only significant in the subepicardial zone when the wine and ethanol drinking groups were compared (Figure 4 and Figure 5).

When considering the pan-macrophage CD68 marker, the differences in densities were nonsignificant between all groups within each zone, except for the borderline significant difference between the wine and ethanol groups in the peri-infarct zone (Figure 4 and Figure 5).

The CD163/CD68 ratio combines these two densities in a single metric providing an insight into the fraction of M2 macrophages within the total macrophage population. It is clearly visible from the boxplots in Figure 5 that the effect on the CD163/CD68 ratio was greatest in the wine group, followed by the ethanol and then the water group. The differences between the groups were significant for all between-group combinations within all zones, except for the nonsignificant difference between the water and ethanol groups in the subepicardial zone. 

**Figure 6:**
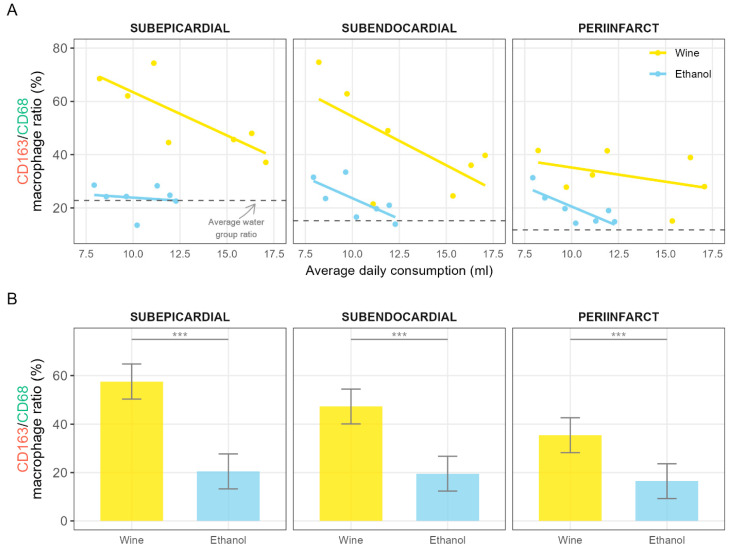
Comparison of CD163/CD68 macrophage ratios between the ethanol and wine drinking groups across different zones. (**A**) The ratio in the trend with respect to the average daily ethanol consumption by animals in each group across analyzed zones. The horizontal dashed line denotes the average value of the ratio for the water control group. (**B**) Comparison of CD163/CD68 macrophage ratios adjusted for the amount of ethanol consumed. The error bars denote the 95% confidence interval. The horizontal lines at the top of the plots and the text above them indicate the significance of Student’s two-sample *t*-tests. *** *p* < 0.001.

Even though the difference between the wine and ethanol drinking groups in terms of average alcoholic beverage consumption was not significant, based on the results from our previous study on the effects of wine and ethanol consumption on survival rate of rats after a MI, we assumed to find a dose-dependent association of CD163/CD68 ratio with the volume of consumed ethanol. Visualizing the relationship, a decreasing trend in the ratios with increasing volumes of consumption was noticed (Figure 6A).

Furthermore, an obvious lack of animals that consumed more than 12.5 mL of ethanol/water solution (more than about 1.6 mL of pure ethanol) was observed. This was due to the fact that such animals did not survive the 96 h post-MI period. Such an upper limit was not present in animals consuming white wine. Consequently, a linear model was used to calculate estimated marginal means by adjusting for the observed difference in maximum value cutoffs (Figure 6B). When these estimated means were compared, the differences in the values of CD163/CD68 ratios between the two groups became even more significant within all three zones (Figure 6B).

## 4. Discussion

The key finding of this study is that the most favorable immunophenotypic profile of macrophages, in the context of the proliferative phase of repair following a surgically induced MI, was observed in rats consuming white wine. This was demonstrated by the highest CD163/CD68 macrophage antigens ratio in the infarct-affected cardiac tissues in the wine group relative to the ethanol group and water drinking controls (Figure 5). Differences between the groups were even more pronounced when the CD163/CD68 ratios were adjusted for the volumes of alcohol consumed by the animals (Figure 6B).

Traditional dichotomic phenotyping involves M1 macrophages that mediate tissue damage and initiate inflammatory responses, and anti-inflammatory M2 macrophages are essential players in the resolution of inflammation. So, M2/M1 balance is associated with the fate and dynamics of recovery of an organ in inflammation or injury [29,30]. The ratio of CD163, an M2-like macrophage marker, and CD68, a pan-macrophage marker, can be used to reflect the M2/M1 ratio [31,32]. The finding that white wine consumption resulted in significantly higher M2/M1 macrophage polarization in all tissue slices of the post-infarcted myocardium in comparison with the ethanol group, indicates that non-alcoholic constituents of white wine may markedly modulate the post-infarction inflammation and healing process. Phenolic compounds are considered the main non-alcoholic bioactive constituents of wine. Because of white wine-making methods, where grape juice ferments without contact with grape skin and seeds, the total phenolic content in standard white wines is much lower than that in red wines [17,33]. Nonetheless, a wide range of phenolic compounds are present in white wine, and some of them in considerable concentrations. These include phenolic acids, particularly hydroxycinnamates, of which caffeic acid is a typical representative [33]. There is also tyrosol, that has been reported to be the second most abundant nonhydroxycinnamate monophenolic in many white wines [34]. Tyrosol in wine is produced during the fermentation process from the yeast metabolism of amino acid tyrosine [35].

Interestingly, it was shown that ethanol may stimulate the endogenous generation of tyrosol in the body and promote tyrosol absorption from different dietary sources [36]. Antioxidant and anti-inflammatory properties of tyrosol and caffeic acid have been proposed by different authors as important contributors to the overall beneficial biological effects of moderate white wine consumption [34,37]. In cardiovascular tissues, caffeic acid and its derivatives have been proven to have a positive effect in ischemia/reperfusion injuries by diminishing cellular dysfunction caused by different physicochemical agents [37,38]. Tyrosol has also been described as a cardioprotective agent against ischemia-induced stress by significantly reducing myocardial infarct size and improving left ventricular myocardial function [39]. Moreover, cardioprotective effects in ischemia/reperfusion injury in rats that were gavaged for 30 days with white wine, in which the main non-alcoholic components were caffeic acid, tyrosol and shikimic acid, were reported [40]. The results of our present, and previous, study on the effects of white wine consumption on survival of rats after MI [18] are in general agreement with the abovementioned studies demonstrating the cardiovascular protection of white wine and its constituents.

Although the mechanisms of action of white wine constituents on macrophage polarization in the post-infarcted myocardium are beyond the scope of this study, there is considerable evidence that antioxidant and anti-inflammatory properties of diet-derived phenolics, including those found in wine may, in conditions with an intense inflammatory component, create a microenvironment that promotes macrophage polarization towards a reparative M2 phenotype.

Specifically, macrophage polarization is a precisely regulated and highly complex process involving different transcriptional epigenetic and post-transcriptional regulatory networks, as well as several signaling pathways, including nuclear factor (erythroid-derived 2)-like 2 (Nrf2) and nuclear factor-κB (NF-κB). These two transcription factors are postulated as major regulators of cellular responses to oxidative stress and inflammation, respectively [41,42,43]. It is well documented that antioxidant and anti-inflammatory properties of phenolic compounds from wine are, to a large extent, mediated through the interaction with Nrf2 and NF-κB signaling pathways [44,45,46,47], thereby providing a direct link with macrophage phenotype kinetics.

Another important finding of this study is that ethanol, either consumed in the form of an ethanol/water solution or in the form of white wine, exhibited opposing effects on the CD163/CD68 macrophage ratio as a function of dosage. Namely, the favorable effects of consumption of 13% v/v ethanol/water solution in comparison with water controls on the CD163/CD68 ratio (Figure 6A) vanished with increasing volumes of consumption. At the level of about 12.5 mL of ethanol/water solution consumption (corresponding to approximately 1.6 mL of pure ethanol), the CD163/CD68 ratio dropped to the average value observed in the water control group (Figure 6A). Moreover, animals that were consuming higher volumes of ethanol are absent because they did not survive the 96 h period after the MI, indicating that the upper limit for the protective effect of ethanol was reached. In contrast to the animals from the ethanol group, the animals that were consuming ethanol in the form of white wine did not reach the water control group ratio values even at levels of wine intake above 17 mL (corresponding to approximately 2.2 mL of pure ethanol), and no upper limit was observed (Figure 6A). These results are in full agreement and corroborate the findings of our preceding study on the effects of ethanol and white wine on survival following MI in the same population of animals as the one that was analyzed in the present study. The survival rates were 11/23 (47.8%), 11/17 (64.7%) and 13/18 (72.2%) for water controls, ethanol and white wine group, respectively [18]. This is closely mirrored by the relative ranking of CD163/CD68 ratio values between the experimental groups. Increased CD163/CD68 ratios at lower amounts of ethanol consumption and decreased ratios at higher volumes of consumption are in line with the biphasic J-shaped relationship between morbidity and mortality risk and alcohol intake that is commonly observed in epidemiological studies examining the association between alcohol and human health [2,48].

Finally, the fact that higher CD163/CD68 ratios were preserved even at higher volumes of ethanol consumed in the form of white wine, point to the protective effects of the non-alcoholic components in white wine versus the damaging effects of ethanol and its toxic metabolites.

### Study Limitations

Neither specific markers for M1 phenotype nor markers for distinguishing subtypes of M2 phenotype were used. Further, this study deals with only one phenomenon of the proliferative phase of infarction recovery, focusing on macrophage differentiation, with a limited possibility of relating it to other processes and mechanisms that simultaneously take place. Finally, possible mechanisms linking white wine consumption and macrophage polarization could only be addressed at a speculative level.

## 5. Conclusions

Our results indicate that non-alcoholic constituents of white wine significantly contribute to its superior effects in the favorable modulation of post-infarction inflammation and healing processes relative to that of ethanol alone. 

It appears that CD163/CD68 ratios, as an indicator of macrophage polarization, might be a useful predictor of recovery and survival after MI, at least under controlled experimental conditions.

## Figures and Tables

**Table 1:** Intake of alcoholic beverages and water in the ethanol, wine and water drinking groups. The bottom row shows *p*-values from a one-way ANOVA when three groups were compared or Student’s two sample *t*-tests of differences when the corresponding parameters were compared between two groups. *** *p* < 0.001; n.s. *p* ≥ 0.05.

Group	Alcoholic Beverage (mL)	Water (mL)	Mass (g)
Wine	12.8 ± 1.3	15.2 ± 2.1	325 ± 14
Ethanol	10.3 ± 0.6	12.1 ± 0.1	321 ± 5
Water	/	37 ± 1	336 ± 5
Significance	n.s.	***	n.s.

## Data Availability

The data presented in this study are available upon reasonable request from the corresponding author.
